# Editorial: Impact of deployment in disaster situations on the mental health of emergency responders

**DOI:** 10.3389/fpsyt.2024.1481901

**Published:** 2024-09-30

**Authors:** Ulrich Wesemann, Ali Sahebi, Hubertus Himmerich

**Affiliations:** ^1^ Department of Psychiatry, Psychotherapy and Psychotraumatology, Bundeswehr Hospital Berlin, Berlin, Germany; ^2^ Department of Medical Emergencies and Health in Disasters and Emergencies, Ilam University of Medical Sciences, Ilam, Iran; ^3^ Non-communicable Diseases Research Center, Ilam University of Medical Sciences, Ilam, Iran; ^4^ Department of Psychological Medicine, Institute of Psychiatry, Psychology & Neuroscience (IoPPN), King’s College London, London, United Kingdom

**Keywords:** emergency responder, military personnel, disaster, mental health, crisis intervention [MeSH], deployment (military), PTSD, risk and resilience factors

The mental health consequences of intentionally engineered catastrophic events for emergency responders can be more severe than those caused by accidents or natural disasters (1, 2). The same mental health risks apply to soldiers during attacks and firefights in foreign military operations (3). Individual preparations and follow-ups have been recommended in order to minimize mental health reactions (4). Differentiation of these mental health effects according to occupational groups, gender, age, event, and other accompanying factors such as spatial and temporal proximity to the event, equipment, tasks performed on site (clearly regulated, professional affiliation, etc.), personal impact, and other factors, are necessary (5, 6).

Various studies have shown that emergency responders from different professions react differently to a major disaster (7, 8). Most studies have focused on post-traumatic stress disorder (PTSD), but the range of mental health effects is far wider. This means that often not only the emergency responders themselves are affected, but the negative effects are also felt in the family relationships (9).

There are numerous publications on the psychological effects of disasters on emergency personnel, but there is comparatively little systematic research on training programs to prevent such negative effects. Therefore, we proposed a Research Topic that covers these aspects and could lead to better pre- and post-deployment training, more individualized crisis intervention, and destigmatization. In this context, Amini et al. evaluated a training program to improve the knowledge, functional skills and attitudes of disaster responders. As the program proved successful in all aspects mentioned, it is a good example of why more efforts should be made to develop and deliver more individualized programs. A similar, but somewhat more specialized approach was taken by Lin et al. They investigated the extent to which psychiatrists are prepared to deal with mental health problems following disasters. They found that although staff are highly experienced and qualified, postgraduate psychiatric training programs in this area still have room for improvement. This applies to the non-pharmacological approaches of dealing with stress-related symptoms.

Ziehr and Merkt describe their concept of strategic resilience as the result of psychological, physical and cognitive resilience. It aims to minimize the weaknesses of the individual constructs by implementing this generalized approach. This multidimensional construct covers different perspectives from military, economic or stress research. This concept can be used by different organizations to develop their own stress management preparation programs. These so-called “problem-oriented interventions” have proven successful in previous evaluations.

A lack of programs designed to strengthen strategic resilience can lead to a higher risk for mental disorders and undesirable behavior. Research is needed to specifically identify such reactions. Zhang et al. investigated nurses in the post-pandemic era. They found mediating effects of low social support and high presenteeism on turnover intention and post-traumatic stress disorder among nurses. These results can be used for targeted prevention work. Social support from employers, adapted working time models and the prevention of presenteeism could all contribute to this. Even if a completely flexible design seems unrealistic for most organizations, adjustments can be made in at least some areas. Muysewinkel et al. showed that good preparation is central to crisis management. This applies not only to the response to terrorist attacks but ultimately to operations in all major disasters. Preparation and training should not only focus on psychological and ethical aspects, but also on performance. The better prepared the emergency services are for crises, the more likely they are to be successful in such situations.

In addition to a lack of social support, Ebrahim et al. found that female gender, direct exposure to critical incidents and alcohol consumption are important risk factors for post-traumatic stress disorder (PTSD) in police officers. Langner et al. found high pre-existing burdens in military personnel as risk factor for developing burnout syndrome post-deployment. Focusing on these aspects prior to deployment could help tailor programs more specifically to the needs of the emergency service personnel.

In summary, our Research Topic covers risk factors, prevention, specific training to improve preparedness and resilience as well as the mental health consequences and their management in emergency responders who are sent to disaster situations. We thank the international group of authors from Belgium, China, Ethiopia, Germany, Iran, Japan and Taiwan for their excellent contributions to this Research Topic. We also thank the reviewers who gave useful feedback and thus helped to improve the manuscripts. Whilst a single Research Topic cannot cover all relevant facets, we hope that this article collection can make a valuable and significant contribution to the field.
